# Pediatric Miller Fisher Syndrome; Characteristic Presentation and Comparison with Adult Miller Fisher Syndrome

**DOI:** 10.3390/jcm9123930

**Published:** 2020-12-03

**Authors:** Yeonji Jang, Jae-Hwan Choi, Jong Hee Chae, Byung Chan Lim, Seong-Joon Kim, Jae Ho Jung

**Affiliations:** 1 Department of Ophthalmology, Seoul National University Children’s Hospital, Seoul National University College of Medicine, 101 Daehak-ro Jongno-gu, Seoul 03080, Korea; ibeyj3721@naver.com (Y.J.); ophjun@snu.ac.kr (S.-J.K.); 2 Department of Neurology, Pusan National Yangsan Hospital, Yangsan 50612, Korea; rachelbolan@hanmail.net; 3 Department of Pediatrics, Pediatric Clinical Neuroscience Center, Seoul National University Children’s Hospital, Seoul National University College of Medicine, Seoul 03080, Korea; chaeped1@snu.ac.kr (J.H.C.); prabbit7@snu.ac.kr (B.C.L.)

**Keywords:** Miller Fisher syndrome, acute inflammatory demyelinating polyneuropathy, ophthalmoplegia, antiganglioside antibodies

## Abstract

Background: We aimed to investigate the characteristic presentation of Miller Fisher syndrome (MFS) in pediatrics and compare it with that in adults. Methods: We performed a retrospective review of medical records, laboratory findings, and disease course of pediatric MFS. The data were compared with those of adult MFS, and literature review was done. Unpaired and paired comparisons between groups were made using Wilcoxon rank-sum and signed-rank tests, respectively. Results: Median age for pediatric MFS was 9.8 ± 6.5 years. There were 5 (45.5%) male and 6 (54.5%) female patients. All patients had preceding infection. Two patients (22.2%) had tested positive for anti-GQ1b antibody. Ten patients (90.1%) were treated with intravenous immunoglobulin, and 2 (18.2%) also received intravenous methylprednisolone. Within one month, 8 (72.7%) patients showed recovery, and all 11 (100%) recovered fully within 3 months. Further, the pediatric group had higher frequency of unilateral involvement of ophthalmoplegia, ataxia, and autonomic symptoms but lower antiganglioside antibody positivity and manifestations of areflexia than the adult group. Conclusions: Neuro-ophthalmic manifestations and disease course of pediatric MFS were similar to those of adult MFS as stated in the literature. However, the presence of autonomic symptoms was higher and anti-GQ1b antibody positivity was lower in pediatric MFS than in adult MFS.

## 1. Introduction

Miller Fisher syndrome (MFS) is an acute self-limiting disorder characterized by a clinical triad of ophthalmoplegia, ataxia, and areflexia [1,2]. MFS has been considered a variant of Guillain-Barré syndrome (GBS) and can overlap with the pharyngeal-cervical-brachial (PCB) variants of GBS, or Bickerstaff brainstem encephalitis (BBE) in the clinical course [3]. Many atypical clinical manifestations, beyond the classic triad, are considered for the differential diagnosis between posterior fossa tumor, Wernicke syndrome, botulism, or meningitides [4].

Because anti-GQ1b ganglioside antibodies have been detected in the sera of patients during the acute phase of MFS, BBE, and GBS with ophthalmoparesis, they seem to have a close relationship with MFS [5]. Therefore, anti-GQ1b antibodies have been considered as a crucial biomarker for MFS.

However, these clinical findings and laboratory results were based on the adult patient group because the prevalence of MFS in pediatric patients is significantly lower than that in adults [6,7]. There are only limited case reports or case series about MFS in pediatric patients. The paucity of information may lead to difficulties in the diagnosis of MFS in the pediatric patient group.

The aim of this study was to describe the distinct clinical characteristics and disease course of MFS in pediatric patients and compare them with those in adult patients.

## 2. Materials and Methods

This retrospective, case–control comparative study included patients diagnosed with MFS at Seoul National University Children’s Hospital (SNUCH) and Pusan National University Yangsan Hospital (PNUYH). The study protocol was reviewed and approved by the Institutional Review Boards of Seoul National University Hospital and Pusan National University Yangsan Hospital. The study procedures were conducted in accordance with the tenets of the Declaration of Helsinki. The requirement for informed consent was waived because of the retrospective nature of the study.

### 2.1. Subjects and Materials

We retrospectively reviewed the medical records of the patients with MFS between January 2001 and December 2019. Pediatric patients were classified as being under 18 years of age at the time of diagnosis. Pediatric MFS patients from SNUCH were included, and adult MFS patients from PNUYH were also included for comparison.

The diagnoses of MFS and subtyping were categorized as follows [2]: (1) classic MFS; ophthalmoplegia and ataxia; (2) incomplete MFS; acute ophthalmoparesis, ataxic neuropathy, ptosis, or mydriasis; (3) Bickerstaff brainstem encephalitis (BBE); ophthalmoplegia, ataxia, and hypersomnolence; (4) overlapping MFS; overlap with Guillain-Barre syndrome (GBS) variants. Furthermore, overlapping MFS was divided into classic GBS, pharyngeal-cervical-brachial weakness (PCB), paraparetic GBS, and bifacial weakness with paranesthesia.

The data in the records revealed that all patients underwent neuro-ophthalmologic examinations including best-corrected visual acuities, presence of ptosis, ocular motility test, presence of internal ophthalmoplegia (pupil palsy), presence of ataxia, and tendon reflex. Further, we collected the data of preceding infection history, presence of anti-GQ1b antibodies, nerve conduction study, cerebrospinal fluid (CSF) analysis, and atypical symptoms or signs beyond the classic MFS symptom triad. We excluded patients who had a previous neurological illness or less than 6 months of follow-up.

### 2.2. Analysis

We investigated clinical features of pediatric MFS and determined its distinct characteristics by a comparison between the pediatric group and the adult group. We compared the clinical manifestations, laboratory findings, and disease course between the pediatric patient group and the adult patient group. The Fisher’s exact test and independent *T*-test were used to compare the results, while Mann–Whitney’s U-test was used in the circumstance that needed a nonparametric test. Statistical analysis was performed using the SPSS program (ver. 20.0; SPSS, Inc., Chicago, IL, USA).

### 2.3. Literature Review

We performed a literature search of the PubMed (Medline) databases for articles published up to March 2020 including the search terms “pediatric”, “children”, and “childhood” in combination with “Miller Fisher syndrome”, “Miller-Fisher syndrome”, and “MFS”. Case reports or series about pediatric MFS were included. Studies written in non-English language or having the full text unavailable were excluded for accuracy. Each article obtained from the search was investigated to determine its potential inclusion in the review.

## 3. Results

### 3.1. Pediatric Miller Fisher Syndrome Characteristics

A total of 11 patients with pediatric MFS were included. The demographic and clinical characteristics of the patients are listed in Table 1. The mean age of MFS onset in the pediatric group was 9.8 ± 6.5 years.

First, all the patients had preceding infection: 5 (45.5%) had gastrointestinal symptoms, 6 (54.5%) had upper respiratory symptoms, and 1 (9.1%) had fever with ear pain. Second, regarding the initial symptoms presented, 7 (63.6%) patients had diplopia, and 4 (36.4%) patients, 1 in each, complained of lower leg weakness, gait disturbance, facial palsy, and voice change. The initial symptoms appeared on average 7.9 ± 4.8 days after the preceding infection.

Third, regarding the clinical diagnosis of the patients, 6 (54.5%) had classic MFS, 4 (36.4%) had overlapping MFS, and 1 (9.1%) had acute ophthalmoparesis. Nine (81.8%) showed atypical clinical manifestations; among them, 3 (27.3%) had autonomic dysfunction (night sweating, hypertension, and tachycardia), 1 (9.1%) complained about urticaria, and 1 (9.1%) had tinnitus.

Fourth, in terms of laboratory test at initial visit, 2 (22.2%) of 9 patients had tested positive for anti-GQ1b antibodies. We excluded 1 patient who was examined for antiganglioside antibody 12 years after the onset. Seven patients (63.6%) had albumin-cytologic dissociation in the cerebrospinal fluid study. In addition, nerve conduction studies demonstrated that 6 (60%) of 10 patients who had available examination results had prolonged terminal latency and decreased nerve conduction velocity.

Last, 10 (90.9%) pediatric patients had been treated with intravenous immunoglobulin, and 2 (18.2%) had also received intravenous dexamethasone injection. One (9.1%) patient received supportive care without medical treatment.

Nine (81.8%) patients had recovered completely within one month, and the remaining 2 (18.2%) recovered within three months.

### 3.2. Comparison between Pediatric Miller Fisher Syndrome and Adult Miller Fisher Syndrome

Table 2 shows the clinical features, laboratory findings, and disease course in the pediatric and the adult MFS. The two groups showed similar clinical features, but pediatric MFS cases tended to have more unilateral involvement of external ophthalmoplegia (*p* = 0.01), ataxia (*p* = 0.01), and autonomic symptoms (*p* = 0.04) than adult MFS cases. Areflexia was a less dominant feature in pediatric MFS than in adult MFS (*p* = 0.04). Regarding laboratory findings, pediatric patients showed lower positivity in anti-GQ1b antibody testing (*p* = 0.02) and higher albumin-cytologic dissociation (*p* < 0.01) than adult patients. The number of pediatric patients who showed complete improvement within a month was higher than that in adult patients *(p* = 0.04).

### 3.3. Literature Review

No case–control or cohort studies on pediatric MFS are available in the literature. Recently, Yoon et al. conducted a retrospective review of anti-GQ1b antibody syndrome in children, but it relied on serologic diagnosis [8]. Otherwise, 53 pediatric cases of 41 case reports were found, and the clinical characteristics of the reviewed cases are demonstrated in Table 3 [9,10,11,12,13,14,15,16,17,18,19,20,21,22,23,24,25,26,27,28,29,30,31,32,33,34,35,36,37,38,39,40,41,42,43,44,45,46,47,48,49]. Details of individual cases were also available as supplement.

## 4. Discussion

This retrospective comparative study showed that neuro-ophthalmic manifestations and disease course of pediatric MFS were similar to those of adult MFS. Pediatric MFS patients had a good prognosis; furthermore, pediatric MFS patients tended to recover faster than adult MFS patients. However, there was a lower incidence of bilateral ocular manifestation at initial presentation among pediatric MFS patients than among adult MFS patients. The presence of autonomic symptoms, such as hypertension, tachycardia, and night sweating, was higher, and anti-GQ1b antibody positivity at the time of diagnosis was lower in pediatric MFS than in adult MFS.

Preceding infection is an important clue for differential diagnosis in MFS. Koga et al. showed in their case–control study that *Campylobacter jejuni* and *Haemophilus influenzae* infections were evident in 21% and 8% of MFS patients, respectively [50]. Berlit and Rakicky reported that 71.8% of MFS patients had a preceding viral infection [51]. Yoon et al. demonstrated that in Korean pediatric MFS cases, 72.7% had a preceding infection, and the majority of them had gastrointestinal symptoms [8]. In the literature review, 84.9% of patients had preceding illness, and upper respiratory infection was the most common infection in pediatric patients (46.7%). Our study showed that 10 (90.9%) patients had preceding gastrointestinal or upper respiratory symptoms. One patient developed MFS after an event of ear pain with fever in this study. Similarly, unusual infections such as acute pyelonephritis, acute otitis media, acute arthritis with viral infection, measles, and mumps were reported [15,27,36,37,40].

Interestingly, in this study, more pediatric MFS patients presented autonomic symptoms than did adult patients. Previously, Malhotra et al. reported that three pediatric patients with MFS showed hypertension [9]. They suggested a possible association between autonomic instability and MFS in pediatrics. The literature review also revealed similar results: seven cases demonstrated autonomic manifestations, primarily hypertension and tachycardia (13.2%, Table 3) [9,23,26,27]. Mori et al. reported that 16% of adult MFS patients showed autonomic symptoms, but they were due to all micturition disturbances [52]. Acute ophthalmoplegia combined with autonomic symptoms, such as hypertension or tachycardia, could be helpful diagnostic clues for pediatric MFS.

In the clinical features of ophthalmoplegia, the prevalence of bilateral involvement at the initial visit in pediatric patients was lower than that in adults. MFS has a progressive pattern in the early phase; the pattern of ophthalmoplegia may therefore differ depending on the timing of diagnosis. For instance, studies have shown six cases starting with unilateral external ophthalmoparesis and then spreading bilaterally as the disease progressed [13,14,16,18,30,41]. The incidence of bilateral involvement in MFS was expected to be part of the disease progression. We therefore infer that the laterality may not be a distinctive feature of pediatric MFS. However, we would like to highlight that progressive ophthalmoplegia after preceding infection could be a helpful feature for MFS diagnosis in children.

Pediatric MFS patients had more ataxia than did adult MFS patients. There was no subtype difference between pediatric and adult cases in our study, although most pediatric MFS patients had the classic MFS. In general, pediatric patients have limitations in expressing their symptoms compared to adult patients. Therefore, we suggest that it might be helpful to closely observe the obvious sign ataxia in MFS diagnosis in pediatrics.

Serological, immunological, and pathological evidence showed the inconclusive role of anti-GQ1b antibodies in MFS [53,54,55,56]. The positivity rate for anti-GQ1b antibodies has been reported as more than 80% in MFS. Therefore, testing for antibodies has been considered as a shred of supportive evidence for the diagnosis of MFS, especially in adult patients [52]. However, only 22.2% tested positive for anti-GQ1b antibodies in our study. The literature review for pediatric MFS case reports revealed that 25 out of 38 cases (65.8%) tested positive for anti-GQ1b antibodies (Table 3). From our investigation and literature review, we postulated that the presence of anti-GQ1b antibodies is lower among pediatrics than among adults. This discrepancy may be explained in the following manner. First, the host-mimicking immune response to ganglioside epitopes may be different in the pediatric group, especially in the early infantile group. In the literature review, only one case presented ganglioside antibody among six patients aged under two years old who tested positive for anti-GQ1b antibodies [9,12,18,19,31,49]. Second, the timing of antibody testing may yield different results; anti-GQ1b IgG antibody titers peak at the onset of the disease, then decay rapidly during the course of clinical recovery [57], eventually becoming undetectable as early as one month after onset [58]. The timing of the testing can determine the sensitivity or specificity of the test results. In our study, six (54.5%) patients had tested for anti-GQ1b antibody within three days of onset, but others had delayed testing. We therefore suggest that anti-GQ1b antibody testing should be performed immediately when MFS is suspected. On the other hand, anti-GQ1b testing may have a limited role in the diagnosis of MFS in pediatrics compared to that in adults.

In this study, seven (63.6%) pediatric patients showed albumin-cytologic dissociation, and it was much higher than that of adult patients. However, we supposed this finding had less meaning than others because several studies reported albumin-cytologic dissociation in MFS, but the dissociation ranged widely from 37% to 76% [55,59,60,61,62]. Furthermore, because normal cerebrospinal fluid protein values decline over the first year of life, albumino-cytologic dissociation of pediatric group might be overestimated [63,64,65].

MFS is generally regarded as a self-limiting, benign condition, and the limited epidemiological evidence available supports this view. The median period between neurologic onset and the disappearance of ataxia and ophthalmoplegia was one and three months, respectively [51,52]. However, this epidemiological evidence is based on data from adult patients. From the current study, pediatric patients also had a good prognosis and similar outcome to adult patients group. In addition, pediatric patients showed a higher percentage of complete recovery within one month than did adult patients (*p* = 0.04).

Our study had some limitations. First, because of the retrospective nature of the study, we had a relatively small number of subjects included as well as variable investigations, descriptions, and follow-up. Second, ganglioside antibody was expressed in different forms, which can be of diagnostic value in atypical MFS. Koga et al. demonstrated the possibility of other ganglioside antibodies rather than GQ1b antibody in seronegative MFS patients [53]; however, we could not test other antiganglioside antibody forms in all patients. Third, it has been reported that the intensity of anti-GQ1b antibody correlated with disease activity [5]. Therefore, further studies for serial antibody testing and other antiganglioside antibody testing can expand our understanding of this unusual disease.

In summary, we observed that pediatric MFS shared many clinical characteristics with adult MFS and had a good prognosis. However, pediatric MFS had several distinct features, accompanied by autonomic symptoms such as hypertension and low positivity of anti-GQ1b antibody.

## Figures and Tables

**Table 1:** Demographic and clinical characteristics of pediatric Miller Fisher syndrome (MFS).

Diagnosis	Age Range/ Sex	Ophthalmoplegia			Ataxia	Hyporeflexia or Areflexia	Additional Manifestations	Ganglioside Antibodies	Timing of Ab Test	CSF/NCS Abnormalities	Treatment	Time to Complete Improvement
		EO	IO	Ptosis								
cMFS	16/M	V(R), H(R)	R	R	(+)	(−)	Dysesthesia, dizziness, night sweat, urticaria	GQ1b	0	(+)/(−)	IVIG	1 month
cMFS	17/M	V(R), H(R)	(−)	R	(+)	(+)	Dysesthesia	(−)	0	(−)/(+)	IVIG	1 month
cMFS	12/F	V(B), H(B)	B	(−)	(+)	(-)	Dysesthesia, ocular pain	(−)	2	(+)/(+)	IVIG	1 month
cMFS	1/M	H(L,ab)	(−)	(−)	(+)	(+)	(−)	No study result	-	(−)/(−)	IVIG, IV steroid	3 months
cMFS	2/M	V(B), H(B)	(−)	B	(+)	(−)	(−)	(−)	5	(−)/(−)	IVIG	1 month
cMFS	13/F	V(L), H(R)	L	(−)	(+)	(+)	Facial palsy, dysesthesia, fever, tachycardia	GQ1b	0	(+)/(+)	IVIG, IV steroid	1 month, recur
oMFS (MFS/GBS/PCB)	2/F	(−)	(−)	B	(+)	(+)	Facial palsy, limb weakness, dysphagia, dysarthria	No study result	-	(+)/(+)	IVIG	3 months
oMFS (AAN/GBS/PCB)	7/F	(−)	(−)	(−)	(+)	(+)	voice change, tongue deviation, limb weakness	(−)	3	(+)/(+)	IVIG	1 month
oMFS (AAN/GBS/PCB)	1/F	(−)	(−)	(−)	(+)	(+)	Limb weakness, dysphagia, hoarseness, hypertension	(−)	8	(+)/(+)	IVIG	1 month
oMFS (MFS/PCB)	15/F	H(B,ab)	(−)	(−)	(+)	(−)	Tinnitus, uvula deviation, absent gag reflex	(−)	18	(−)/no study result	Supportive care	1 month
AO	17/M	V(L), H(L)	(−)	L	(−)	(−)	Lower limb pain	(−)	1	(+)/(−)	IVIG	3 months

cMFS = classic MFS; oMFS = overlapping MFS; GBS = Guillain-Barre syndrome; PCB = pharyngeal-cervical-brachial weakness; AAN = acute ataxic neuropathy; AO = acute ophthalmoparesis; EO = external ophthalmoplegia; IO = internal ophthalmoplegia; Ab = antibodies; CSF = cerebrospinal fluid; NCS = nerve conduction study; B = bilateral; R = right eye; L = left eye; ab = abduction limitation only; IVIG = intravenous immunoglobulin.

**Table 2:** Comparison between pediatric and adult Miller Fisher syndrome.

	Pediatric (*n* = 11)	Adult (*n* = 36)	*p*-Value	
Age of onset	9.8 ± 6.5	46.4 ± 16.9	
Male:Female	5:6	22:14	0.4895
Preceding infection	11/11 (100%)	31/36 (86.1%)	0.3216
External ophthalmoplegia	9/11 (81.8%)	35/36 (97.2%)	0.1323
Internal ophthalmoplegia	3/11 (27.3%)	13/36 (36.1%)	0.7252
Unilateral:Bilateral	4:5	2:34	0.0103 *
Ataxia	10/11 (90.9%)	17/36 (47.2%)	0.0141 *
Areflexia	6/11 (54.5%)	31/36 (86.1%)	0.0394 *
Dysesthesia	5/11 (45.5%)	12/36 (33.3%)	0.4933
Multiple cranial nerve	3/11 (27.3%)	13/36 (36.1%)	0.7252
Limb weakness	3/11 (27.3%)	4/36 (11.1%)	0.3296
Autonomic symptoms	3/11 (27.3%)	1/36 (2.8%)	0.0352 *
Antiganglioside Antibody positivity	2/9 ^a^ (22.2%)	25/35 ^a^ (71.4%)	0.0172 *
Albumin-cytologic dissociation in CSF study	7/11 (63.6%)	4/32 ^a^ (12.5%)	0.0022 *
NCS abnormality	6/10 ^a^ (60.0%)	6/18 ^a^ (33.3%)	0.2425
Treatment			
Supportive care	1/11 (9.1%)	8/36 (22.2%)	0.6631
IVIG only	8/11 (72.7%)	26/36 (72.2%)	1
IVIG + PP or systemic corticosteroid	2/11 (18.2%) ^b^	2/36 (5.6%) ^c^	0.2294
Response to treatment			
Within 1 month	8/11 (72.7%)	13/36 (36.1%)	0.0433 *
Within 3 months	11/11 (100%)	29/36 (80.6%)	0.1751

^a.^ Statistics using the number of patients who were available for each test. ^b^ Two patients received IVIG + steroid. ^c^ One patient received IVIG + steroid, and one patient received IVIG + plasmapheresis * *p* < 0.05, CSF = cerebrospinal fluid; NCS = nerve conduction study; IVIG = intravenous immunoglobulin; PP = plasmapheresis.

**Table 3:** Demographics of pediatric Miller Fisher syndrome in the literature, and its comparison with pediatric MFS in our study.

	Published Data (*n* = 53)	Present Study (*n* = 11)
Age of onset	6.9 ± 4.2	9.8 ± 6.5
Male:Female	28:22	5:6
Preceding infection	45/53 (84.9%)	11/11 (100%)
External ophthalmoplegia	52/53 (98.1%)	9/11 (81.8%)
Internal ophthalmoplegia	21/53 (39.6%)	3/11 (27.3%)
Unilateral:Bilateral	9 ^a^:44	4:5
Ataxia	39/53 (73.6%)	10/11 (90.9%)
Areflexia	41/53 (77.4%)	6/11 (54.5%)
Autonomic symptoms	7/53 (13.2%)	3/11 (27.3%)
Anti-GQ1b Antibody positivity	25/38 ^b^ (65.8%)	2/9 ^b^ (22.2%)
Antiganglioside Antibody positivity rather than GQ1b ^c^	8/38 ^b^ (21.1%)	0/9 ^b^ (0%)
Albumin-cytologic dissociation in CSF study	18/49 ^b^ (36.7%)	7/11 (63.6%)
NCS abnormality	13/31 ^b^ (41.9%)	6/10 ^b^ (60.0%)
Treatment		
Supportive care	12/53 (22.6%)	1/11 (9.1%)
IVIG only	30/53 (56.6%)	8/11 (72.7%)
IVIG + PP or systemic corticosteroid	7/53 (13.2%)	2/11 (18.2%)
Other treatments ^d^	4/53 (7.5%)	0/11 (0%)
Response to treatment		
Within 1 month	12/34 ^b^ (35.3%)	8/11 (72.7%)
Within 3 months	26/34 ^b^ (76.5%)	11/11 (100%)

^a.^ Unilateral involvement at disease onset. ^b^ Statistics using the number of patients who were available for each test. ^c^ GM, GD, GT antibody. ^d^ Only corticosteroid treatment (intravenous or oral).

## Supplementary Materials

The following are available online at https://www.mdpi.com/2077-0383/9/12/3930/s1, Table S1: Published cases of pediatric Miller-Fisher syndrome.
