# Supplementary material for: Pediatric Miller Fisher Syndrome; Characteristic Presentation and Comparison with Adult Miller Fisher Syndrome
Source: J Clin Med. 2020 Dec 3;9(12):3930. doi: 10.3390/jcm9123930 (PMC7761853; doi:10.3390/jcm9123930)
Supplement: Supplementary file 1 [file jcm-09-03930-s001.pdf]

Table S1. Published cases of pediatric Miller-Fisher syndrome.

Patient	Age/ Sex	Preceding infection	Ophthalmoplegia			Ataxia	Hyporeflexia	Extra-triad manifestations	Ganglioside antibodies	CSF/NCS abnormalities	Treatment	Recovery	etc	Ref.
			EO	IO	Ptosis									
1	2/F	URI	(-)	(-)	B	(+)	(+)	Optic nerve, chiasm enhancement, hypertension	(-)	/	IVIG			[9]
2	11/F	GI	L	(-)	(-)	(+)	(+)	Vomiting, urinary retention, constipation, headache, hypertension, disc edema, optic chiasm enhancement	/	(+)/	IVIG			[9]
3	17/F	Fever, headache	(-)	(-)	(-)	(+)	(+)	Facial palsy, headache, nausea, vomiting, hypertension	/	(+)/	IVIG			[9]
4	14/M	EBV	B	(-)	B	(-)	(+)	Facial palsy, tongue deviation, absent gag reflex, dysarthria, dysphagia	GQ1b, GT1a, GT1b	(+)/(+)	IVIG	6mo		[10]
5	12/M	fever	B	B	B	(+)	(+)	Dysarthria, hoarseness, facial palsy, convergence paresis	GQ1b	(-)/(-)	IVIG	2mo	recur	[11]
6	8/M	fever	B	(-)	B	(+)	(+)	Dysphagia, dysarthria, facial palsy	GQ1b	(-)/(-)	IVIG, IV steroid	1mo	recur	[11]
7	2/M	fever	B(ab)	(-)	R	(+)	(+)	(-)	(-)	(-)/	IVIG	2wk		[12]
8	4/F	(-)	B	(-)	B	(+)	(+)	(-)	(-)	(-)/	IVIG	3wk		[12]
9	6/?	(-)	L→B [a]	(-)	B	(+)	(+)	Drowsiness, dysphagia, hypopnea, facial palsy	GQ1b	(-)/	IVIG			[13]
10	2/M	URI	L→B [a]	B	B	(+)	(+)	Facial palsy, respiratory distress	/	(-)/	IVIG	6mo		[14]
11	10/F	APN	V(R), H(B)	(-)	(-)	(+)	(+)	Facial palsy, limb weakness	(-)	/	IVIG	2mo	E.coli	[15]
12	13/M	GI	L→B [a]	L	L→B	(+)	(+)	(-)	(-)	(-)/(-)	IVIG	3mo		[16]
13	10/M	GI	Diplopia [b]	L	L	(+)	(-)	Headache, dizziness, dysarthria	GQ1b	(-)/	IVIG	4mo	E.coli	[17]

14	9/F	GI	L(ab)→B [a]	(-)	(-)	(+)	(+)	Headache, nausea	GQ1b	(-)/(-)	IVIG			[18]
15	2/F	(-)	B(ab)	(-)	(-)	(+)	(+)	Dysarthria, dysphagia	(-)	(-)/(+)	IVIG, IV steroid	4wk		[18]
16	11/F	URI	B	(-)	(-)	(+)	(+)	Soft palate paresis	GQ1b	(-)/(+) [c]	IVIG			[18]
17	2/F	URI	B	(-)	B	(+)	(+)	(-)	(-)	(+)/	IVIG	2mo		[19]
18	11m/?	URI	B	(-)	B	(+)	(+)	(-)	/	(+)/(+)	IVIG	1wk		[20]
19	16m/?	URI	B(ab)	(-)	L	(+)	(+)	(-)	/	(+)/(+)	conservative	2mo	preterm	[20]
20	6/F	URI	B	B	B	(+)	(+)	Facial palsy, dysarthria, dysphagia	GQ1b	(+)/	IVIG	3mo		[21]
21	10/M	GI	B	B	B	(+)	(+)	Headache, nausea, vomiting, neck stiffness, lower limb pain, soft palate paresis, dysarthria, dysphagia, hoarseness	GT1a, GQ1b	(+)/(+)	conservative	2mo		[22]
22	5/F	URI	B	B	B	(+)	(+)	Vomiting, drowsiness, respiratory distress, hypertension, sinus tachycardia	GQ1b	(+)/(+)	IVIG, IV steroid	5wk		[23]
23	4/M	URI	B	B	B	(+)	(+)	Headache, upper lid retraction, nystagmus, limb weakness, facial palsy	/	(-)/	IVIG			[24]
24	4/M	URI	B	B	B	(+)	(+)	Hypersomnolence, intentional tremor	GQ1b	(+)/(-)	IVIG			[25]
25	7/M	(-)	B(ab)→B [a]	(-)	(-)	(+)	(+)	Hypohidrosis	/	(-)/(+)	IVIG, IV steroid	2mo		[26]
26	3/M	AOM	B	B	B	(+)	(+)	Drowsiness, headache, nausea, vomiting, facial palsy	GQ1b	(+)/	IV steroid, IVIG	3mo		[27]
27	11/M	URI	B	B	(-)	(+)	(+)	Hypertension	GQ1b	(-)/	IVIG			[28]
28	3/M	fever	B	L	(-)	(-)	(+)	Hypertension, tachycardia, respiratory distress, tonic-clonic seizure, limb weakness	/	(-)/(+)	IVIG, PP		VZV	[29]
29	7/M	URI	B	(-)	B	(+)	(+)	Facial palsy	GQ1b	(+)/(+)	IVIG	2mo		[30]
30	9/F	GI	L(ab)→B [a]	(-)	(-)	(+)	(+)	Headache, nausea, vomiting	GQ1b	(-)/(-)	IVIG	3mo		[31]

31	2/F	(-)	B(ab)	(-)	(-)	(+)	(-)	Vomiting, dysarthria, dysphagia	(-)	(-)/(+)	IVIG, IV steroid	4wk		[31]
32	3/M	URI	B	B	B	(+)	(+)	Headache, limb weakness, intention tremor, dysesthesia, dysarthria	/	(+)/(-)	IVIG			[32]
33	6/F	URI	B(ab)	(-)	B	(+)	(+)	Dysarthria	/	(+)/(-)	IVIG	4wk	M.pneu moniae	[33]
34	5/F	(-)	B	B	B	(+)	(-)	Convergence paresis, facial palsy, dysphagia, dysarthria, nystagmus, intention tremor, generalized hypotonia	/	(-)/(-)	ACTH, IV steroid			[34]
35	5/M	(-)	B	(-)	B	(+)	(+)	Dysphagia, dysarthria, facial weakness, generalized hypotonia, intention tremor	/	/(+)	conservative			[34]
36	14/M	URI	B(ab)	(-)	(-)	(-)	(+)	Nystagmus, convergence paresis,	(-)	(-)/(-)	IVIG	1wk	recur	[35]
37	9/F	URI (measles)	B	B	B	(+)	(+)	Absent gag reflex, agitation, disorientation, stupor, respiratory distress	/	(+)/(-)	conservative	6mo		[36]
38	22m/F	URI (mumps)	B	B	B	(+)	(+)	Nystagmus, generalized hypotonia, hypersomnolence	/	(+)/	IV steroid	6mo		[37]
39	5/M	GI	B(ab)	(-)	(-)	(+)	(+)	(-)	GQ1b	(+)/	IVIG			[38]
40	7/M	GI	B	(-)	B	(-)	(+)	Decreased visual acuities, nystagmus, convergence paresis, pallesthesia	GA1	(-)/(-)	conservative			[39]
41	5/F	Fever, joint pain	B	B	(-)	(-)	(-)	(-)	/	/	PO steroid		CHIKV	[40]
42	6/M	GI	B(ab)	(-)	(-)	(-)	(-)	(-)	GQ1b, GT1a, GD3	(-)/(-)	conservative			[41]
43	4/M	GI	L→B [a]	(-)	(-)	(-)	(-)	(-)	GQ1b, GT1a, GM2	(-)/(-)	conservative			[41]
44	10/F	URI	L	L	L	(-)	(-)	(-)	GQ1b, GM1	(-)/	PO steroid	2mo		[42]
45	14/M	GI	B	B	(-)	(-)	(-)	Headache, vomiting, facial palsy	GQ1b	(-)/(-)	conservative	4mo		[43]

46	13/F	GI	B [d]	(-)	(-)	(+)	(+)	Dysarthria, dysphagia	(-)	(-)/(-)	IVIG	4wk		[44]
47	11/F	(-)	B	(-)	(-)	(+)	(+)	Limb weakness	(-)	(+)/	IVIG	1mo		[45]
48	3/M	URI	B(ab)→B [a]	(-)	(-)	(+)	(+)	Dysarthria, drowsiness, fever, respiratory distress, facial palsy	GM1	(-)/(-)	conservative			[46]
49	5/F	URI	B	B	B	(-)	(-)	(-)	GQ1b	(-)/	IVIG	4mo		[47]
50	11/M	GI	B	B	B	(-)	(-)	(-)	GQ1b	(-)/	conservative	8mo		[48]
51	14/M	GI	B(ab)	(-)	(-)	(-)	(-)	(-)	GQ1b, GM1, GD1a, GD1b	(-)/	conservative	2mo		[48]
52	3/M	GI	B	(-)	R	(-)	(-)	(-)	GQ1b	(-)/(-)	conservative	1mo		[48]
53	2/M	URI	(-)	(-)	B	(-)	(+)	(-)	GQ1b	(-)/(+)	IVIG	1wk		[49]

[a] In these cases, patients had started with unilateral ophthalmoplegia or abduction palsy only, and then aggravated to bilateral ophthalmoplegia as disease progression.

[b] In this case, only the presence of diplopia was recorded without evaluating the ophthalmoplegia. [c] In this case, MNCV, SNCV and F waves were normal, but H-reflexes were absent. [d] Gaze palsy, left. EO = external ophthalmoplegia; IO = internal ophthalmoplegia; CSF = cerebrospinal fluid = NCS = nerve conduction study; URI = upper respiratory infection; IVIG = intravenous immunoglobulin; GI = gastrointestinal infection; APN = acute pyelonephritis; AOM = acute otitis media; PP = plasmapheresis; ACTH = adrenocorticotropic hormone; CHIKV = Chikungunya virus; B = bilateral; R = right eye; L = left eye; ab = abduction limitation only; MNCV = motor nerve conduction velocity; SNCV = sensory nerve conduction velocity; mo = month; wk = week.
